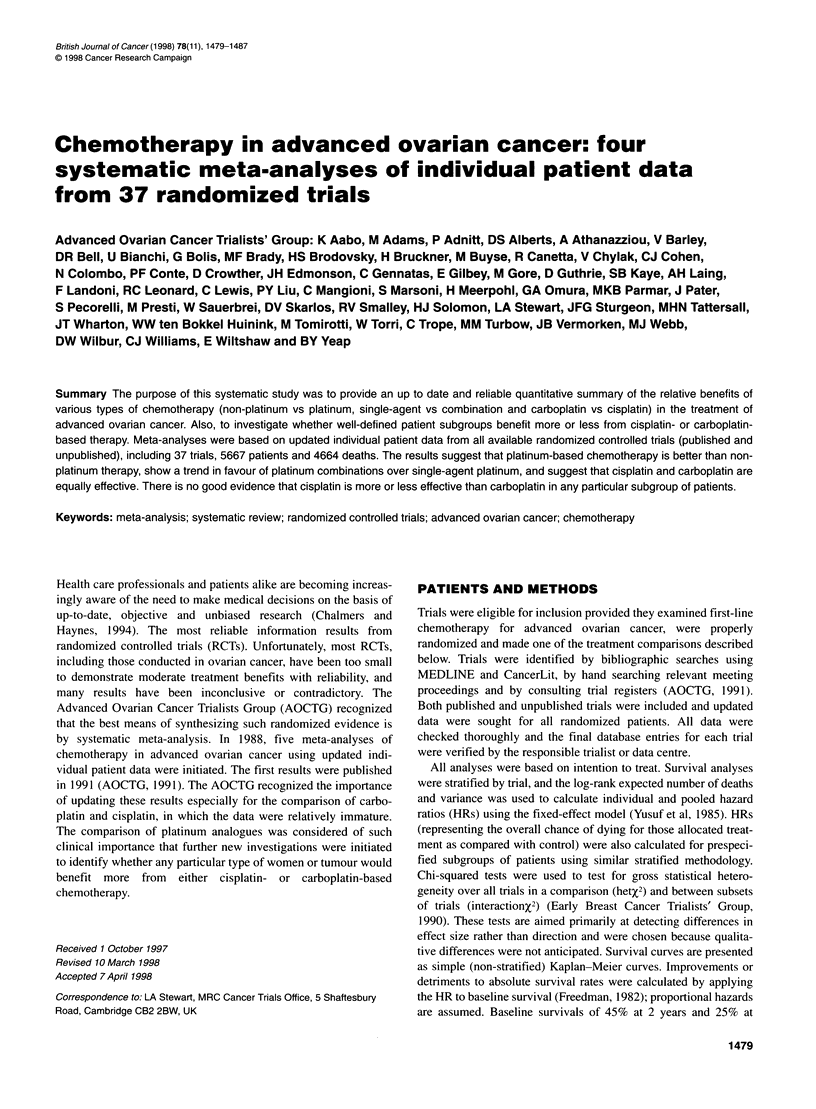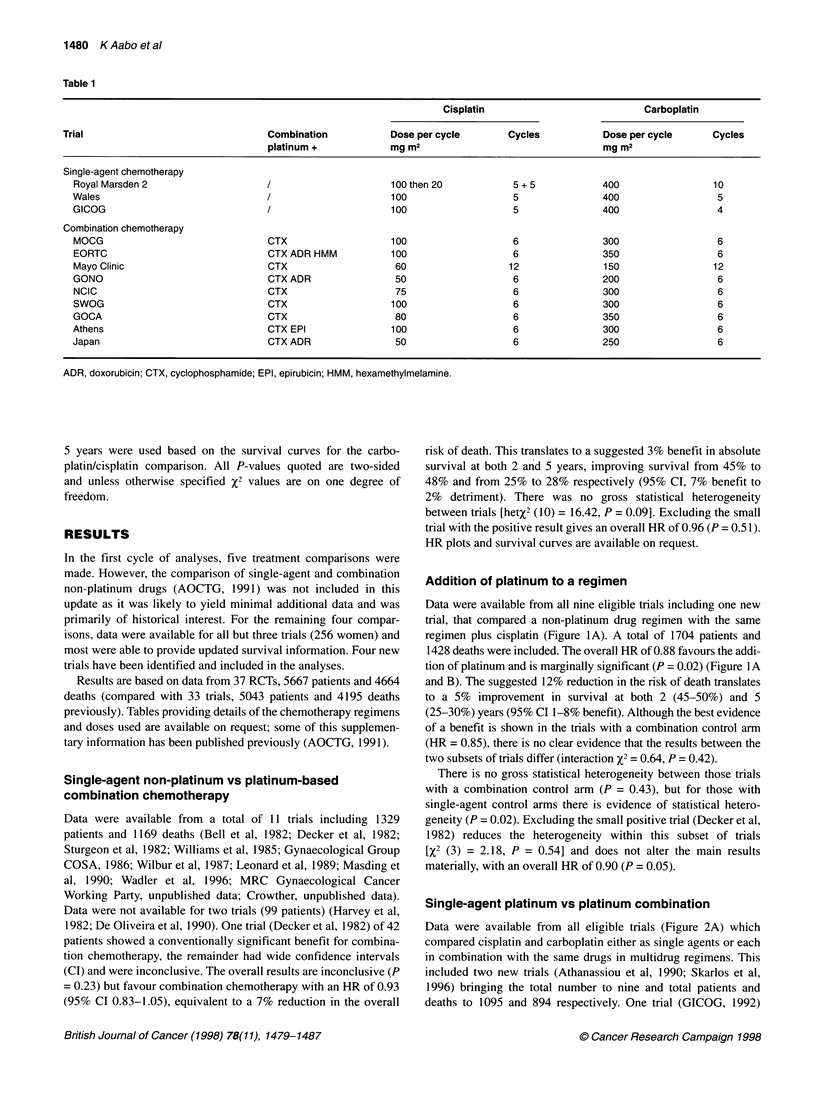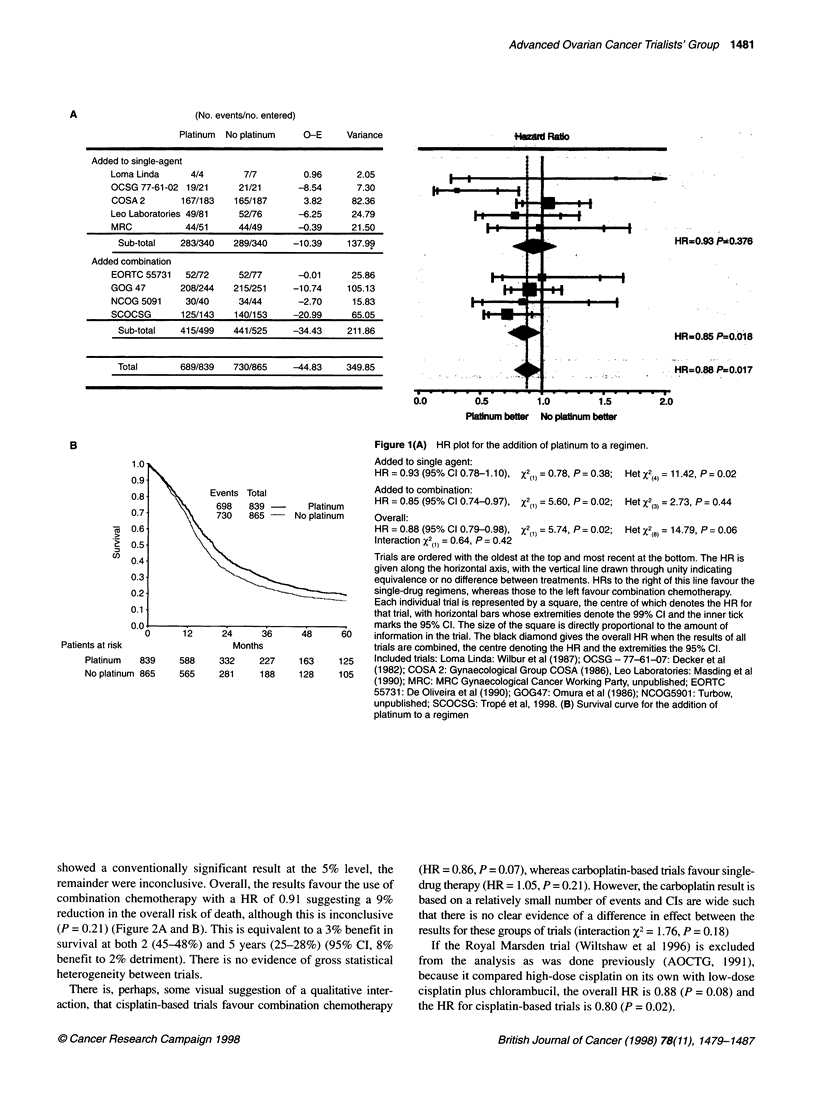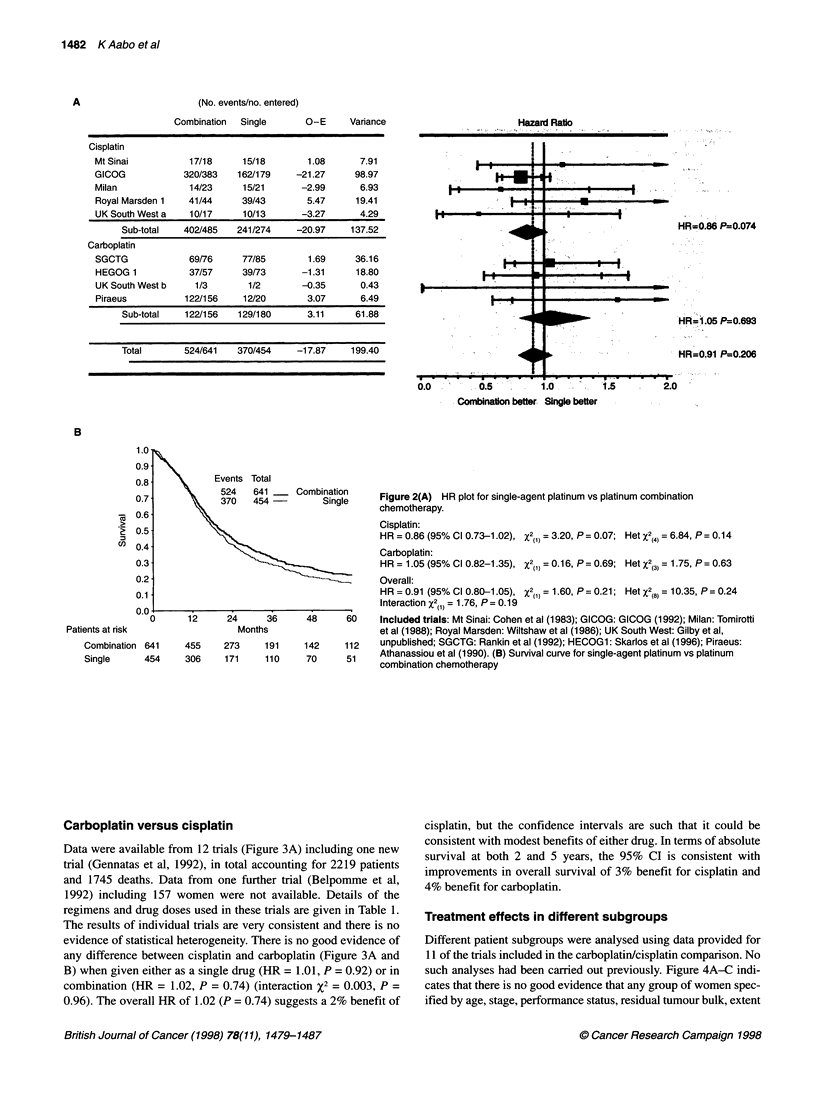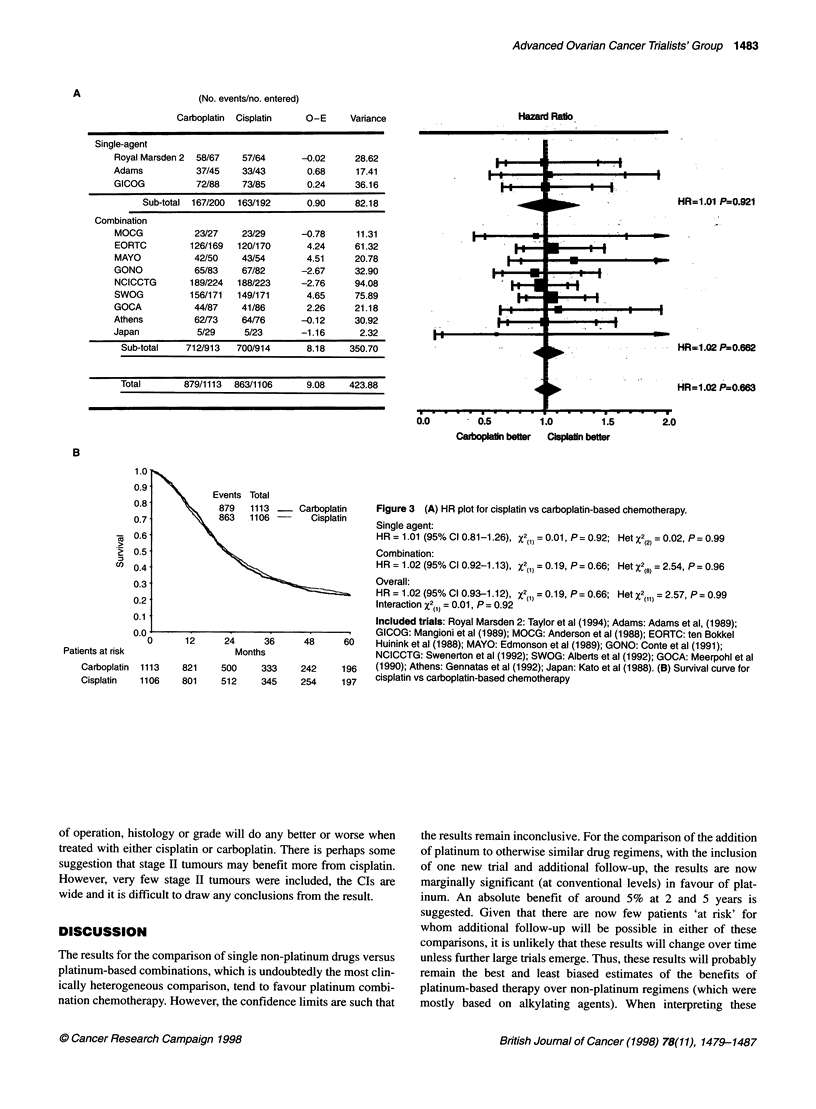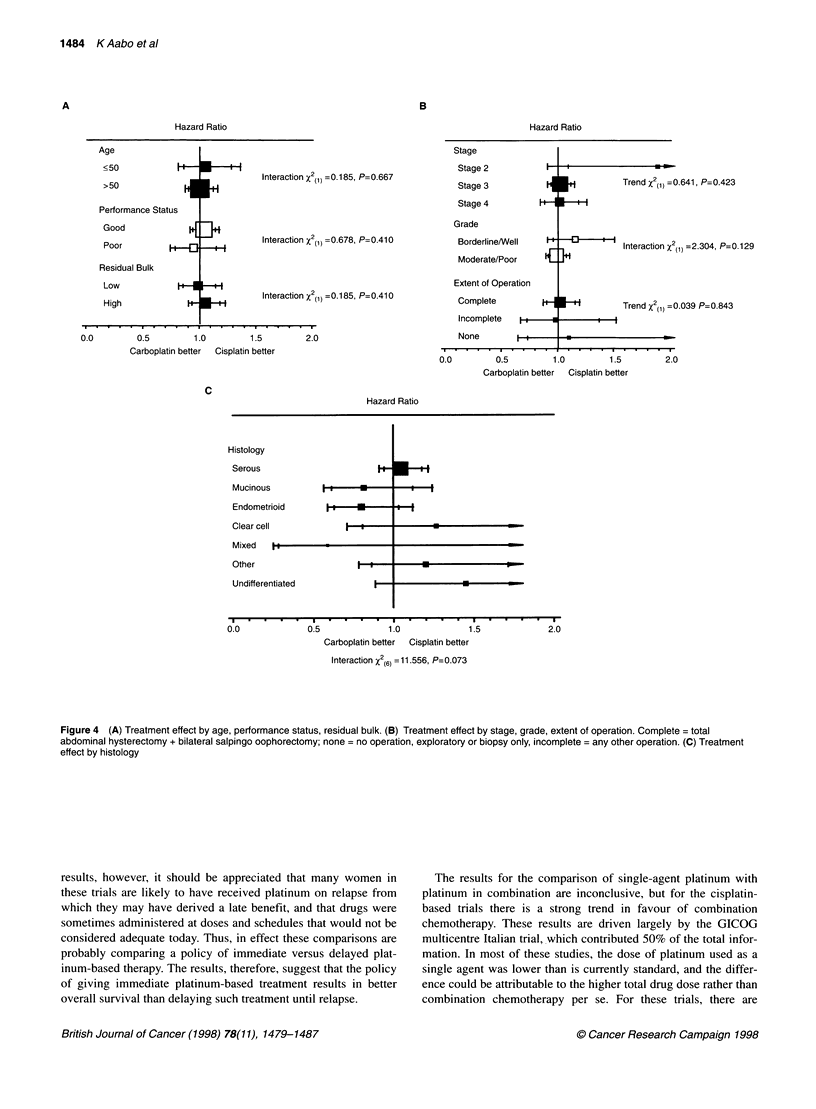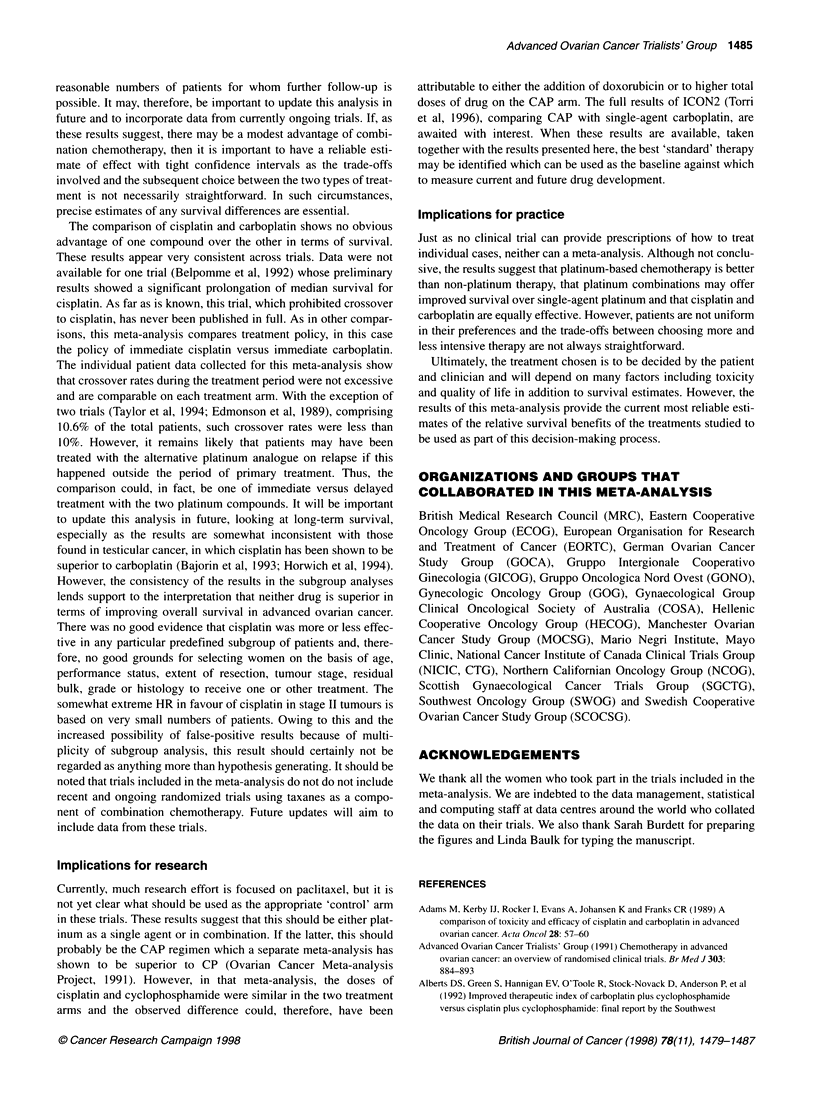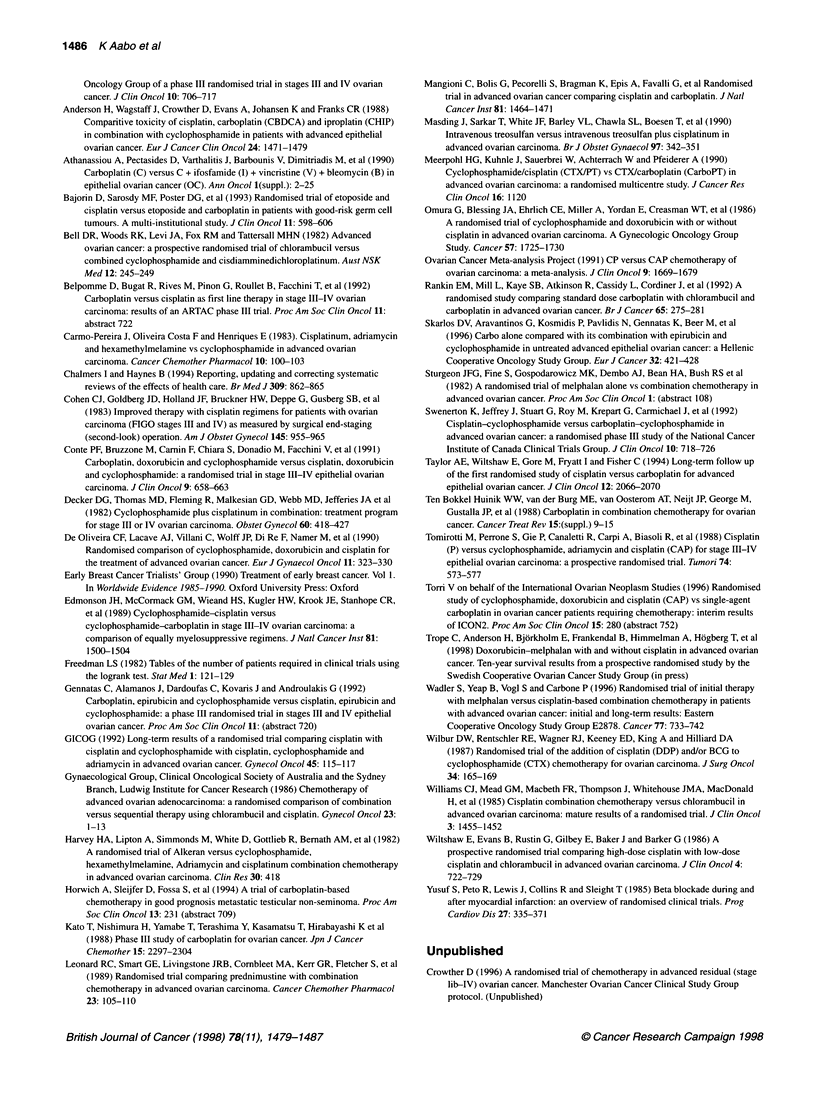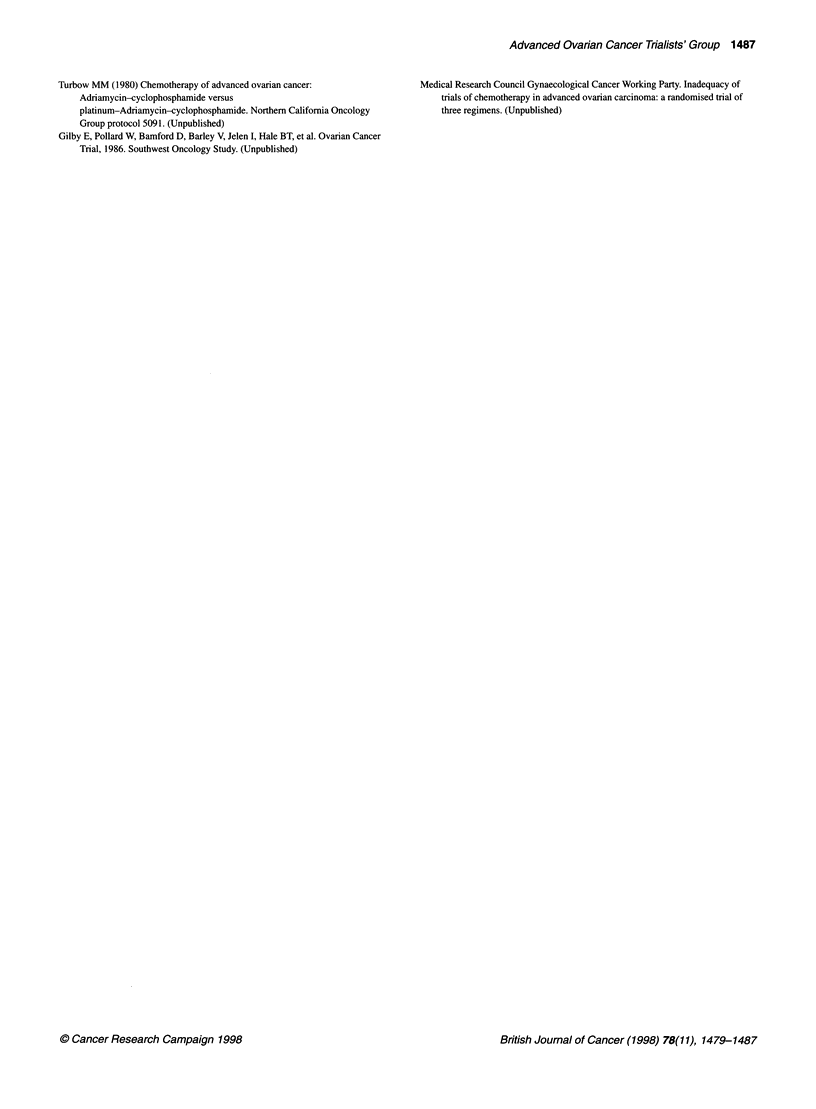# Chemotherapy in advanced ovarian cancer: four systematic meta-analyses of individual patient data from 37 randomized trials. Advanced Ovarian Cancer Trialists' Group.

**DOI:** 10.1038/bjc.1998.710

**Published:** 1998-12

**Authors:** K. Aabo, M. Adams, P. Adnitt, D. S. Alberts, A. Athanazziou, V. Barley, D. R. Bell, U. Bianchi, G. Bolis, M. F. Brady, H. S. Brodovsky, H. Bruckner, M. Buyse, R. Canetta, V. Chylak, C. J. Cohen, N. Colombo, P. F. Conte, D. Crowther, J. H. Edmonson, C. Gennatas, E. Gilbey, M. Gore, D. Guthrie, B. Y. Yeap

## Abstract

**Images:**


British Journal of Cancer (1998) 78(11), 1479–1487
© 1998 Cancer Research Campaign

### Chemotherapy in advanced ovarian cancer: four systematic meta-analyses of individual patient data from 37 randomized trials

**Advanced Ovarian Cancer Trialists' Group: K Aabo, M Adams, P Adnitt, DS Alberts, A Athanazziou, V Barley, DR Bell, U Bianchi, G Bolis, MF Brady, HS Brodovsky, H Bruckner, M Buyse, R Canetta, V Chylak, CJ Cohen, N Colombo, PF Conte, D Crowther, JH Edmonson, C Gennatas, E Gilbey, M Gore, D Guthrie, SB Kaye, AH Laing, F Landoni, RC Leonard, C Lewis, PY Liu, C Mangioni, S Marsoni, H Meerpohl, GA Omura, MKB Parmar, J Pater, S Pecorelli, M Presti, W Sauerbrei, DV Skarlos, RV Smalley, HJ Solomon, LA Stewart, JFG Sturgeon, MHN Tattersall, JT Wharton, WW ten Bokkel Huinink, M Tomirotti, W Torri, C Trope, MM Turbow, JB Vermorken, MJ Webb, DW Wilbur, CJ Williams, E Wiltshaw and BY Yeap**

**Summary** The purpose of this systematic study was to provide an up to date and reliable quantitative summary of the relative benefits of various types of chemotherapy (non-platinum vs platinum, single-agent vs combination and carboplatin vs cisplatin) in the treatment of advanced ovarian cancer. Also, to investigate whether well-defined patient subgroups benefit more or less from cisplatin- or carboplatin-based therapy. Meta-analyses were based on updated individual patient data from all available randomized controlled trials (published and unpublished), including 37 trials, 5667 patients and 4664 deaths. The results suggest that platinum-based chemotherapy is better than non-platinum therapy, show a trend in favour of platinum combinations over single-agent platinum, and suggest that cisplatin and carboplatin are equally effective. There is no good evidence that cisplatin is more or less effective than carboplatin in any particular subgroup of patients.

**Keywords:** meta-analysis; systematic review; randomized controlled trials; advanced ovarian cancer; chemotherapy

Health care professionals and patients alike are becoming increasingly aware of the need to make medical decisions on the basis of up-to-date, objective and unbiased research (Chalmers and Haynes, 1994). The most reliable information results from randomized controlled trials (RCTs). Unfortunately, most RCTs, including those conducted in ovarian cancer, have been too small to demonstrate moderate treatment benefits with reliability, and many results have been inconclusive or contradictory. The Advanced Ovarian Cancer Trialists Group (AOCTG) recognized that the best means of synthesizing such randomized evidence is by systematic meta-analysis. In 1988, five meta-analyses of chemotherapy in advanced ovarian cancer using updated individual patient data were initiated. The first results were published in 1991 (AOCTG, 1991). The AOCTG recognized the importance of updating these results especially for the comparison of carboplatin and cisplatin, in which the data were relatively immature. The comparison of platinum analogues was considered of such clinical importance that further new investigations were initiated to identify whether any particular type of women or tumour would benefit more from either cisplatin- or carboplatin-based chemotherapy.

*Received 1 October 1997*
*Revised 10 March 1998*
*Accepted 7 April 1998*

*Correspondence to:* LA Stewart, MRC Cancer Trials Office, 5 Shaftesbury Road, Cambridge CB2 2BW, UK

### PATIENTS AND METHODS

Trials were eligible for inclusion provided they examined first-line chemotherapy for advanced ovarian cancer, were properly randomized and made one of the treatment comparisons described below. Trials were identified by bibliographic searches using MEDLINE and CancerLit, by hand searching relevant meeting proceedings and by consulting trial registers (AOCTG, 1991). Both published and unpublished trials were included and updated data were sought for all randomized patients. All data were checked thoroughly and the final database entries for each trial were verified by the responsible trialist or data centre.

All analyses were based on intention to treat. Survival analyses were stratified by trial, and the log-rank expected number of deaths and variance was used to calculate individual and pooled hazard ratios (HRs) using the fixed-effect model (Yusuf et al, 1985). HRs (representing the overall chance of dying for those allocated treatment as compared with control) were also calculated for prespecified subgroups of patients using similar stratified methodology. Chi-squared tests were used to test for gross statistical heterogeneity over all trials in a comparison (het$\chi^2$) and between subsets of trials (interaction$\chi^2$) (Early Breast Cancer Trialists' Group, 1990). These tests are aimed primarily at detecting differences in effect size rather than direction and were chosen because qualitative differences were not anticipated. Survival curves are presented as simple (non-stratified) Kaplan–Meier curves. Improvements or detriments to absolute survival rates were calculated by applying the HR to baseline survival (Freedman, 1982); proportional hazards are assumed. Baseline survivals of 45% at 2 years and 25% at

**Table 1**

| Trial | Combination platinum + | Cisplatin Dose per cycle mg m$^2$ | Cisplatin Cycles | Carboplatin Dose per cycle mg m$^2$ | Carboplatin Cycles |
|---|---|---|---|---|---|
| Single-agent chemotherapy | | | | | |
| Royal Marsden 2 | / | 100 then 20 | 5 + 5 | 400 | 10 |
| Wales | / | 100 | 5 | 400 | 5 |
| GICOG | / | 100 | 5 | 400 | 4 |
| Combination chemotherapy | | | | | |
| MOCG | CTX | 100 | 6 | 300 | 6 |
| EORTC | CTX ADR HMM | 100 | 6 | 350 | 6 |
| Mayo Clinic | CTX | 60 | 12 | 150 | 12 |
| GONO | CTX ADR | 50 | 6 | 200 | 6 |
| NCIC | CTX | 75 | 6 | 300 | 6 |
| SWOG | CTX | 100 | 6 | 300 | 6 |
| GOCA | CTX | 80 | 6 | 350 | 6 |
| Athens | CTX EPI | 100 | 6 | 300 | 6 |
| Japan | CTX ADR | 50 | 6 | 250 | 6 |

ADR, doxorubicin; CTX, cyclophosphamide; EPI, epirubicin; HMM, hexamethylmelamine.

5 years were used based on the survival curves for the carboplatin/cisplatin comparison. All *P*-values quoted are two-sided and unless otherwise specified $\chi^2$ values are on one degree of freedom.

### RESULTS

In the first cycle of analyses, five treatment comparisons were made. However, the comparison of single-agent and combination non-platinum drugs (AOCTG, 1991) was not included in this update as it was likely to yield minimal additional data and was primarily of historical interest. For the remaining four comparisons, data were available for all but three trials (256 women) and most were able to provide updated survival information. Four new trials have been identified and included in the analyses.

Results are based on data from 37 RCTs, 5667 patients and 4664 deaths (compared with 33 trials, 5043 patients and 4195 deaths previously). Tables providing details of the chemotherapy regimens and doses used are available on request; some of this supplementary information has been published previously (AOCTG, 1991).

### Single-agent non-platinum vs platinum-based combination chemotherapy

Data were available from a total of 11 trials including 1329 patients and 1169 deaths (Bell et al, 1982; Decker et al, 1982; Sturgeon et al, 1982; Williams et al, 1985; Gynaecological Group COSA, 1986; Wilbur et al, 1987; Leonard et al, 1989; Masding et al, 1990; Wadler et al, 1996; MRC Gynaecological Cancer Working Party, unpublished data; Crowther, unpublished data). Data were not available for two trials (99 patients) (Harvey et al, 1982; De Oliveira et al, 1990). One trial (Decker et al, 1982) of 42 patients showed a conventionally significant benefit for combination chemotherapy, the remainder had wide confidence intervals (CI) and were inconclusive. The overall results are inconclusive ($P$ = 0.23) but favour combination chemotherapy with an HR of 0.93 (95% CI 0.83–1.05), equivalent to a 7% reduction in the overall risk of death. This translates to a suggested 3% benefit in absolute survival at both 2 and 5 years, improving survival from 45% to 48% and from 25% to 28% respectively (95% CI, 7% benefit to 2% detriment). There was no gross statistical heterogeneity between trials [het$\chi^2$ (10) = 16.42, $P$ = 0.09]. Excluding the small trial with the positive result gives an overall HR of 0.96 ($P$ = 0.51). HR plots and survival curves are available on request.

### Addition of platinum to a regimen

Data were available from all nine eligible trials including one new trial, that compared a non-platinum drug regimen with the same regimen plus cisplatin (Figure 1A). A total of 1704 patients and 1428 deaths were included. The overall HR of 0.88 favours the addition of platinum and is marginally significant ($P$ = 0.02) (Figure 1A and B). The suggested 12% reduction in the risk of death translates to a 5% improvement in survival at both 2 (45–50%) and 5 (25–30%) years (95% CI 1–8% benefit). Although the best evidence of a benefit is shown in the trials with a combination control arm (HR = 0.85), there is no clear evidence that the results between the two subsets of trials differ (interaction $\chi^2$ = 0.64, $P$ = 0.42).

There is no gross statistical heterogeneity between those trials with a combination control arm ($P$ = 0.43), but for those with single-agent control arms there is evidence of statistical heterogeneity ($P$ = 0.02). Excluding the small positive trial (Decker et al, 1982) reduces the heterogeneity within this subset of trials [$\chi^2$ (3) = 2.18, $P$ = 0.54] and does not alter the main results materially, with an overall HR of 0.90 ($P$ = 0.05).

### Single-agent platinum vs platinum combination

Data were available from all eligible trials (Figure 2A) which compared cisplatin and carboplatin either as single agents or each in combination with the same drugs in multidrug regimens. This included two new trials (Athanassiou et al, 1990; Skarlos et al, 1996) bringing the total number to nine and total patients and deaths to 1095 and 894 respectively. One trial (GICOG, 1992)

 *© Cancer Research Campaign 1998*

**A**

| | (No. events/no. entered) | | | |
|---|---|---|---|---|
| | Platinum | No platinum | O–E | Variance |
| Added to single-agent | | | | |
| Loma Linda | 4/4 | 7/7 | 0.96 | 2.05 |
| OCSG 77-61-02 | 19/21 | 21/21 | –8.54 | 7.30 |
| COSA 2 | 167/183 | 165/187 | 3.82 | 82.36 |
| Leo Laboratories | 49/81 | 52/76 | –6.25 | 24.79 |
| MRC | 44/51 | 44/49 | –0.39 | 21.50 |
| Sub-total | 283/340 | 289/340 | –10.39 | 137.99 |
| Added combination | | | | |
| EORTC 55731 | 52/72 | 52/77 | –0.01 | 25.86 |
| GOG 47 | 208/244 | 215/251 | –10.74 | 105.13 |
| NCOG 5091 | 30/40 | 34/44 | -2.70 | 15.83 |
| SCOCSG | 125/143 | 140/153 | –20.99 | 65.05 |
| Sub-total | 415/499 | 441/525 | –34.43 | 211.86 |
| Total | 689/839 | 730/865 | –44.83 | 349.85 |

**B**

| Patients at risk | 0 | 12 | 24 | 36 | 48 | 60 |
|---|---|---|---|---|---|---|
| Platinum | 839 | 588 | 332 | 227 | 163 | 125 |
| No platinum | 865 | 565 | 281 | 188 | 128 | 105 |

**Figure 1(A)** HR plot for the addition of platinum to a regimen.

Added to single agent:

HR = 0.93 (95% CI 0.78–1.10), $\chi^2_{(1)} = 0.78$, $P = 0.38$; Het $\chi^2_{(4)} = 11.42$, $P = 0.02$

Added to combination:

HR = 0.85 (95% CI 0.74–0.97), $\chi^2_{(1)} = 5.60$, $P = 0.02$; Het $\chi^2_{(3)} = 2.73$, $P = 0.44$

Overall:

HR = 0.88 (95% CI 0.79–0.98), $\chi^2_{(1)} = 5.74$, $P = 0.02$; Het $\chi^2_{(8)} = 14.79$, $P = 0.06$

Interaction $\chi^2_{(1)} = 0.64$, $P = 0.42$

Trials are ordered with the oldest at the top and most recent at the bottom. The HR is given along the horizontal axis, with the vertical line drawn through unity indicating equivalence or no difference between treatments. HRs to the right of this line favour the single-drug regimens, whereas those to the left favour combination chemotherapy. Each individual trial is represented by a square, the centre of which denotes the HR for that trial, with horizontal bars whose extremities denote the 99% CI and the inner tick marks the 95% CI. The size of the square is directly proportional to the amount of information in the trial. The black diamond gives the overall HR when the results of all trials are combined, the centre denoting the HR and the extremities the 95% CI. Included trials: Loma Linda: Wilbur et al (1987); OCSG – 77–61–07: Decker et al (1982); COSA 2: Gynaecological Group COSA (1986), Leo Laboratories: Masding et al (1990); MRC: MRC Gynaecological Cancer Working Party, unpublished; EORTC 55731: De Oliveira et al (1990); GOG47: Omura et al (1986); NCOG5901: Turbow, unpublished; SCOCSG: Tropé et al, 1998. (**B**) Survival curve for the addition of platinum to a regimen showed a conventionally significant result at the 5% level, the remainder were inconclusive. Overall, the results favour the use of combination chemotherapy with a HR of 0.91 suggesting a 9% reduction in the overall risk of death, although this is inconclusive ($P = 0.21$) (Figure 2A and B). This is equivalent to a 3% benefit in survival at both 2 (45–48%) and 5 years (25–28%) (95% CI, 8% benefit to 2% detriment). There is no evidence of gross statistical heterogeneity between trials.

There is, perhaps, some visual suggestion of a qualitative interaction, that cisplatin-based trials favour combination chemotherapy (HR = 0.86, $P = 0.07$), whereas carboplatin-based trials favour single-drug therapy (HR = 1.05, $P = 0.21$). However, the carboplatin result is based on a relatively small number of events and CIs are wide such that there is no clear evidence of a difference in effect between the results for these groups of trials (interaction $\chi^2 = 1.76$, $P = 0.18$)

If the Royal Marsden trial (Wiltshaw et al 1996) is excluded from the analysis as was done previously (AOCTG, 1991), because it compared high-dose cisplatin on its own with low-dose cisplatin plus chlorambucil, the overall HR is 0.88 ($P = 0.08$) and the HR for cisplatin-based trials is 0.80 ($P = 0.02$).

*© Cancer Research Campaign 1998*

**A**

| | (No. events/no. entered) | | | |
|---|---|---|---|---|
| | Combination | Single | O–E | Variance |
| Cisplatin | | | | |
| Mt Sinai | 17/18 | 15/18 | 1.08 | 7.91 |
| GICOG | 320/383 | 162/179 | –21.27 | 98.97 |
| Milan | 14/23 | 15/21 | –2.99 | 6.93 |
| Royal Marsden 1 | 41/44 | 39/43 | 5.47 | 19.41 |
| UK South West a | 10/17 | 10/13 | –3.27 | 4.29 |
| Sub-total | 402/485 | 241/274 | –20.97 | 137.52 |
| Carboplatin | | | | |
| SGCTG | 69/76 | 77/85 | 1.69 | 36.16 |
| HEGOG 1 | 37/57 | 39/73 | –1.31 | 18.80 |
| UK South West b | 1/3 | 1/2 | –0.35 | 0.43 |
| Piraeus | 122/156 | 12/20 | 3.07 | 6.49 |
| Sub-total | 122/156 | 129/180 | 3.11 | 61.88 |
| Total | 524/641 | 370/454 | –17.87 | 199.40 |

**B**

| Patients at risk | 0 | 12 | 24 | 36 | 48 | 60 |
|---|---|---|---|---|---|---|
| Combination | 641 | 455 | 273 | 191 | 142 | 112 |
| Single | 454 | 306 | 171 | 110 | 70 | 51 |

**Figure 2(A)** HR plot for single-agent platinum vs platinum combination chemotherapy.

Cisplatin:

HR = 0.86 (95% CI 0.73–1.02), $\chi^2_{(1)} = 3.20$, $P = 0.07$; Het $\chi^2_{(4)} = 6.84$, $P = 0.14$

Carboplatin:

HR = 1.05 (95% CI 0.82–1.35), $\chi^2_{(1)} = 0.16$, $P = 0.69$; Het $\chi^2_{(3)} = 1.75$, $P = 0.63$

Overall:

HR = 0.91 (95% CI 0.80–1.05), $\chi^2_{(1)} = 1.60$, $P = 0.21$; Het $\chi^2_{(8)} = 10.35$, $P = 0.24$

Interaction $\chi^2_{(1)} = 1.76$, $P = 0.19$

**Included trials**: Mt Sinai: Cohen et al (1983); GICOG: GICOG (1992); Milan: Tomirotti et al (1988); Royal Marsden: Wiltshaw et al (1986); UK South West: Gilby et al, unpublished; SGCTG: Rankin et al (1992); HECOG1: Skarlos et al (1996); Piraeus: Athanassiou et al (1990). (**B**) Survival curve for single-agent platinum vs platinum combination chemotherapy

### Carboplatin versus cisplatin

Data were available from 12 trials (Figure 3A) including one new trial (Gennatas et al, 1992), in total accounting for 2219 patients and 1745 deaths. Data from one further trial (Belpomme et al, 1992) including 157 women were not available. Details of the regimens and drug doses used in these trials are given in Table 1. The results of individual trials are very consistent and there is no evidence of statistical heterogeneity. There is no good evidence of any difference between cisplatin and carboplatin (Figure 3A and B) when given either as a single drug (HR = 1.01, $P = 0.92$) or in combination (HR = 1.02, $P = 0.74$) (interaction $\chi^2 = 0.003$, $P = 0.96$). The overall HR of 1.02 ($P = 0.74$) suggests a 2% benefit of cisplatin, but the confidence intervals are such that it could be consistent with modest benefits of either drug. In terms of absolute survival at both 2 and 5 years, the 95% CI is consistent with improvements in overall survival of 3% benefit for cisplatin and 4% benefit for carboplatin.

### Treatment effects in different subgroups

Different patient subgroups were analysed using data provided for 11 of the trials included in the carboplatin/cisplatin comparison. No such analyses had been carried out previously. Figure 4A–C indicates that there is no good evidence that any group of women specified by age, stage, performance status, residual tumour bulk, extent

© Cancer Research Campaign 1998

**A**

| | (No. events/no. entered) | | | |
|---|---|---|---|---|
| | Carboplatin | Cisplatin | O–E | Variance |
| Single-agent | | | | |
| Royal Marsden 2 | 58/67 | 57/64 | –0.02 | 28.62 |
| Adams | 37/45 | 33/43 | 0.68 | 17.41 |
| GICOG | 72/88 | 73/85 | 0.24 | 36.16 |
| Sub-total | 167/200 | 163/192 | 0.90 | 82.18 |
| Combination | | | | |
| MOCG | 23/27 | 23/29 | –0.78 | 11.31 |
| EORTC | 126/169 | 120/170 | 4.24 | 61.32 |
| MAYO | 42/50 | 43/54 | 4.51 | 20.78 |
| GONO | 65/83 | 67/82 | –2.67 | 32.90 |
| NCICCTG | 189/224 | 188/223 | –2.76 | 94.08 |
| SWOG | 156/171 | 149/171 | 4.65 | 75.89 |
| GOCA | 44/87 | 41/86 | 2.26 | 21.18 |
| Athens | 62/73 | 64/76 | –0.12 | 30.92 |
| Japan | 5/29 | 5/23 | –1.16 | 2.32 |
| Sub-total | 712/913 | 700/914 | 8.18 | 350.70 |
| Total | 879/1113 | 863/1106 | 9.08 | 423.88 |

**B**

| Patients at risk | 0 | 12 | 24 | 36 | 48 | 60 |
|---|---|---|---|---|---|---|
| Carboplatin | 1113 | 821 | 500 | 333 | 242 | 196 |
| Cisplatin | 1106 | 801 | 512 | 345 | 254 | 197 |

**Figure 3** (**A**) HR plot for cisplatin vs carboplatin-based chemotherapy.
Single agent:
HR = 1.01 (95% CI 0.81–1.26), $\chi^2_{(1)} = 0.01$, $P = 0.92$; Het $\chi^2_{(2)} = 0.02$, $P = 0.99$
Combination:
HR = 1.02 (95% CI 0.92–1.13), $\chi^2_{(1)} = 0.19$, $P = 0.66$; Het $\chi^2_{(8)} = 2.54$, $P = 0.96$
Overall:
HR = 1.02 (95% CI 0.93–1.12), $\chi^2_{(1)} = 0.19$, $P = 0.66$; Het $\chi^2_{(11)} = 2.57$, $P = 0.99$
Interaction $\chi^2_{(1)} = 0.01$, $P = 0.92$
**Included trials**: Royal Marsden 2: Taylor et al (1994); Adams: Adams et al, (1989); GICOG: Mangioni et al (1989); MOCG: Anderson et al (1988); EORTC: ten Bokkel Huinink et al (1988); MAYO: Edmonson et al (1989); GONO: Conte et al (1991); NCICCTG: Swenerton et al (1992); SWOG: Alberts et al (1992); GOCA: Meerpohl et al (1990); Athens: Gennatas et al (1992); Japan: Kato et al (1988). (**B**) Survival curve for cisplatin vs carboplatin-based chemotherapy of operation, histology or grade will do any better or worse when treated with either cisplatin or carboplatin. There is perhaps some suggestion that stage II tumours may benefit more from cisplatin. However, very few stage II tumours were included, the CIs are wide and it is difficult to draw any conclusions from the result.

### DISCUSSION

The results for the comparison of single non-platinum drugs versus platinum-based combinations, which is undoubtedly the most clinically heterogeneous comparison, tend to favour platinum combination chemotherapy. However, the confidence limits are such that the results remain inconclusive. For the comparison of the addition of platinum to otherwise similar drug regimens, with the inclusion of one new trial and additional follow-up, the results are now marginally significant (at conventional levels) in favour of platinum. An absolute benefit of around 5% at 2 and 5 years is suggested. Given that there are now few patients 'at risk' for whom additional follow-up will be possible in either of these comparisons, it is unlikely that these results will change over time unless further large trials emerge. Thus, these results will probably remain the best and least biased estimates of the benefits of platinum-based therapy over non-platinum regimens (which were mostly based on alkylating agents). When interpreting these

© Cancer Research Campaign 1998

**Figure 4** (**A**) Treatment effect by age, performance status, residual bulk. (**B**) Treatment effect by stage, grade, extent of operation. Complete = total abdominal hysterectomy + bilateral salpingo oophorectomy; none = no operation, exploratory or biopsy only, incomplete = any other operation. (**C**) Treatment effect by histology results, however, it should be appreciated that many women in these trials are likely to have received platinum on relapse from which they may have derived a late benefit, and that drugs were sometimes administered at doses and schedules that would not be considered adequate today. Thus, in effect these comparisons are probably comparing a policy of immediate versus delayed platinum-based therapy. The results, therefore, suggest that the policy of giving immediate platinum-based treatment results in better overall survival than delaying such treatment until relapse.

The results for the comparison of single-agent platinum with platinum in combination are inconclusive, but for the cisplatin-based trials there is a strong trend in favour of combination chemotherapy. These results are driven largely by the GICOG multicentre Italian trial, which contributed 50% of the total information. In most of these studies, the dose of platinum used as a single agent was lower than is currently standard, and the difference could be attributable to the higher total drug dose rather than combination chemotherapy per se. For these trials, there are

 *© Cancer Research Campaign 1998*

reasonable numbers of patients for whom further follow-up is possible. It may, therefore, be important to update this analysis in future and to incorporate data from currently ongoing trials. If, as these results suggest, there may be a modest advantage of combination chemotherapy, then it is important to have a reliable estimate of effect with tight confidence intervals as the trade-offs involved and the subsequent choice between the two types of treatment is not necessarily straightforward. In such circumstances, precise estimates of any survival differences are essential.

The comparison of cisplatin and carboplatin shows no obvious advantage of one compound over the other in terms of survival. These results appear very consistent across trials. Data were not available for one trial (Belpomme et al, 1992) whose preliminary results showed a significant prolongation of median survival for cisplatin. As far as is known, this trial, which prohibited crossover to cisplatin, has never been published in full. As in other comparisons, this meta-analysis compares treatment policy, in this case the policy of immediate cisplatin versus immediate carboplatin. The individual patient data collected for this meta-analysis show that crossover rates during the treatment period were not excessive and are comparable on each treatment arm. With the exception of two trials (Taylor et al, 1994; Edmonson et al, 1989), comprising 10.6% of the total patients, such crossover rates were less than 10%. However, it remains likely that patients may have been treated with the alternative platinum analogue on relapse if this happened outside the period of primary treatment. Thus, the comparison could, in fact, be one of immediate versus delayed treatment with the two platinum compounds. It will be important to update this analysis in future, looking at long-term survival, especially as the results are somewhat inconsistent with those found in testicular cancer, in which cisplatin has been shown to be superior to carboplatin (Bajorin et al, 1993; Horwich et al, 1994). However, the consistency of the results in the subgroup analyses lends support to the interpretation that neither drug is superior in terms of improving overall survival in advanced ovarian cancer. There was no good evidence that cisplatin was more or less effective in any particular predefined subgroup of patients and, therefore, no good grounds for selecting women on the basis of age, performance status, extent of resection, tumour stage, residual bulk, grade or histology to receive one or other treatment. The somewhat extreme HR in favour of cisplatin in stage II tumours is based on very small numbers of patients. Owing to this and the increased possibility of false-positive results because of multiplicity of subgroup analysis, this result should certainly not be regarded as anything more than hypothesis generating. It should be noted that trials included in the meta-analysis do not do not include recent and ongoing randomized trials using taxanes as a component of combination chemotherapy. Future updates will aim to include data from these trials.

### Implications for research

Currently, much research effort is focused on paclitaxel, but it is not yet clear what should be used as the appropriate 'control' arm in these trials. These results suggest that this should be either platinum as a single agent or in combination. If the latter, this should probably be the CAP regimen which a separate meta-analysis has shown to be superior to CP (Ovarian Cancer Meta-analysis Project, 1991). However, in that meta-analysis, the doses of cisplatin and cyclophosphamide were similar in the two treatment arms and the observed difference could, therefore, have been attributable to either the addition of doxorubicin or to higher total doses of drug on the CAP arm. The full results of ICON2 (Torri et al, 1996), comparing CAP with single-agent carboplatin, are awaited with interest. When these results are available, taken together with the results presented here, the best 'standard' therapy may be identified which can be used as the baseline against which to measure current and future drug development.

### Implications for practice

Just as no clinical trial can provide prescriptions of how to treat individual cases, neither can a meta-analysis. Although not conclusive, the results suggest that platinum-based chemotherapy is better than non-platinum therapy, that platinum combinations may offer improved survival over single-agent platinum and that cisplatin and carboplatin are equally effective. However, patients are not uniform in their preferences and the trade-offs between choosing more and less intensive therapy are not always straightforward.

Ultimately, the treatment chosen is to be decided by the patient and clinician and will depend on many factors including toxicity and quality of life in addition to survival estimates. However, the results of this meta-analysis provide the current most reliable estimates of the relative survival benefits of the treatments studied to be used as part of this decision-making process.

### ORGANIZATIONS AND GROUPS THAT COLLABORATED IN THIS META-ANALYSIS

British Medical Research Council (MRC), Eastern Cooperative Oncology Group (ECOG), European Organisation for Research and Treatment of Cancer (EORTC), German Ovarian Cancer Study Group (GOCA), Gruppo Intergionale Cooperativo Ginecologia (GICOG), Gruppo Oncologica Nord Ovest (GONO), Gynecologic Oncology Group (GOG), Gynaecological Group Clinical Oncological Society of Australia (COSA), Hellenic Cooperative Oncology Group (HECOG), Manchester Ovarian Cancer Study Group (MOCSG), Mario Negri Institute, Mayo Clinic, National Cancer Institute of Canada Clinical Trials Group (NICIC, CTG), Northern Californian Oncology Group (NCOG), Scottish Gynaecological Cancer Trials Group (SGCTG), Southwest Oncology Group (SWOG) and Swedish Cooperative Ovarian Cancer Study Group (SCOCSG).

### ACKNOWLEDGEMENTS

We thank all the women who took part in the trials included in the meta-analysis. We are indebted to the data management, statistical and computing staff at data centres around the world who collated the data on their trials. We also thank Sarah Burdett for preparing the figures and Linda Baulk for typing the manuscript.

### REFERENCES

Adams M, Kerby IJ, Rocker I, Evans A, Johansen K and Franks CR (1989) A comparison of toxicity and efficacy of cisplatin and carboplatin in advanced ovarian cancer. *Acta Oncol* **28**: 57–60

Advanced Ovarian Cancer Trialists' Group (1991) Chemotherapy in advanced ovarian cancer: an overview of randomised clinical trials. *Br Med J* **303**: 884–893

Alberts DS, Green S, Hannigan EV, O'Toole R, Stock-Novack D, Anderson P, et al (1992) Improved therapeutic index of carboplatin plus cyclophosphamide versus cisplatin plus cyclophosphamide: final report by the Southwest

© Cancer Research Campaign 1998 

Oncology Group of a phase III randomised trial in stages III and IV ovarian cancer. *J Clin Oncol* **10**: 706–717

Anderson H, Wagstaff J, Crowther D, Evans A, Johansen K and Franks CR (1988) Comparitive toxicity of cisplatin, carboplatin (CBDCA) and iproplatin (CHIP) in combination with cyclophosphamide in patients with advanced epithelial ovarian cancer. *Eur J Cancer Clin Oncol* **24**: 1471–1479

Athanassiou A, Pectasides D, Varthalitis J, Barbounis V, Dimitriadis M, et al (1990) Carboplatin (C) versus C + ifosfamide (I) + vincristine (V) + bleomycin (B) in epithelial ovarian cancer (OC). *Ann Oncol* **1**(suppl.): 2–25

Bajorin D, Sarosdy MF, Poster DG, et al (1993) Randomised trial of etoposide and cisplatin versus etoposide and carboplatin in patients with good-risk germ cell tumours. A multi-institutional study. *J Clin Oncol* **11**: 598–606

Bell DR, Woods RK, Levi JA, Fox RM and Tattersall MHN (1982) Advanced ovarian cancer: a prospective randomised trial of chlorambucil versus combined cyclophosphamide and cisdiamminedichloroplatinum. *Aust NSK Med* **12**: 245–249

Belpomme D, Bugat R, Rives M, Pinon G, Roullet B, Facchini T, et al (1992) Carboplatin versus cisplatin as first line therapy in stage III–IV ovarian carcinoma: results of an ARTAC phase III trial. *Proc Am Soc Clin Oncol* **11**: abstract 722

Carmo-Pereira J, Oliveira Costa F and Henriques E (1983). Cisplatinum, adriamycin and hexamethylmelamine vs cyclophosphamide in advanced ovarian carcinoma. *Cancer Chemother Pharmacol* **10**: 100–103

Chalmers I and Haynes B (1994) Reporting, updating and correcting systematic reviews of the effects of health care. *Br Med J* **309**: 862–865

Cohen CJ, Goldberg JD, Holland JF, Bruckner HW, Deppe G, Gusberg SB, et al (1983) Improved therapy with cisplatin regimens for patients with ovarian carcinoma (FIGO stages III and IV) as measured by surgical end-staging (second-look) operation. *Am J Obstet Gynecol* **145**: 955–965

Conte PF, Bruzzone M, Carnin F, Chiara S, Donadio M, Facchini V, et al (1991) Carboplatin, doxorubicin and cyclophosphamide versus cisplatin, doxorubicin and cyclophosphamide: a randomised trial in stage III–IV epithelial ovarian carcinoma. *J Clin Oncol* **9**: 658–663

Decker DG, Thomas MD, Fleming R, Malkesian GD, Webb MD, Jefferies JA et al (1982) Cyclophosphamide plus cisplatinum in combination: treatment program for stage III or IV ovarian carcinoma. *Obstet Gynecol* **60**: 418–427

De Oliveira CF, Lacave AJ, Villani C, Wolff JP, Di Re F, Namer M, et al (1990) Randomised comparison of cyclophosphamide, doxorubicin and cisplatin for the treatment of advanced ovarian cancer. *Eur J Gynaecol Oncol* **11**: 323–330

Early Breast Cancer Trialists' Group (1990) Treatment of early breast cancer. Vol 1. In *Worldwide Evidence 1985–1990*. Oxford University Press: Oxford

Edmonson JH, McCormack GM, Wieand HS, Kugler HW, Krook JE, Stanhope CR, et al (1989) Cyclophosphamide–cisplatin versus cyclophosphamide–carboplatin in stage III–IV ovarian carcinoma: a comparison of equally myelosuppressive regimens. *J Natl Cancer Inst* **81**: 1500–1504

Freedman LS (1982) Tables of the number of patients required in clinical trials using the logrank test. *Stat Med* **1**: 121–129

Gennatas C, Alamanos J, Dardoufas C, Kovaris J and Androulakis G (1992) Carboplatin, epirubicin and cyclophosphamide versus cisplatin, epirubicin and cyclophosphamide: a phase III randomised trial in stages III and IV epithelial ovarian cancer. *Proc Am Soc Clin Oncol* **11**: (abstract 720)

GICOG (1992) Long-term results of a randomised trial comparing cisplatin with cisplatin and cyclophosphamide with cisplatin, cyclophosphamide and adriamycin in advanced ovarian cancer. *Gynecol Oncol* **45**: 115–117

Gynaecological Group, Clinical Oncological Society of Australia and the Sydney Branch, Ludwig Institute for Cancer Research (1986) Chemotherapy of advanced ovarian adenocarcinoma: a randomised comparison of combination versus sequential therapy using chlorambucil and cisplatin. *Gynecol Oncol* **23**: 1–13

Harvey HA, Lipton A, Simmonds M, White D, Gottlieb R, Bernath AM, et al (1982) A randomised trial of Alkeran versus cyclophosphamide, hexamethylmelamine, Adriamycin and cisplatinum combination chemotherapy in advanced ovarian carcinoma. *Clin Res* **30**: 418

Horwich A, Sleijfer D, Fossa S, et al (1994) A trial of carboplatin-based chemotherapy in good prognosis metastatic testicular non-seminoma. *Proc Am Soc Clin Oncol* **13**: 231 (abstract 709)

Kato T, Nishimura H, Yamabe T, Terashima Y, Kasamatsu T, Hirabayashi K et al (1988) Phase III study of carboplatin for ovarian cancer. *Jpn J Cancer Chemother* **15**: 2297–2304

Leonard RC, Smart GE, Livingstone JRB, Cornbleet MA, Kerr GR, Fletcher S, et al (1989) Randomised trial comparing prednimustine with combination chemotherapy in advanced ovarian carcinoma. *Cancer Chemother Pharmacol* **23**: 105–110

Mangioni C, Bolis G, Pecorelli S, Bragman K, Epis A, Favalli G, et al Randomised trial in advanced ovarian cancer comparing cisplatin and carboplatin. *J Natl Cancer Inst* **81**: 1464–1471

Masding J, Sarkar T, White JF, Barley VL, Chawla SL, Boesen T, et al (1990) Intravenous treosulfan versus intravenous treosulfan plus cisplatinum in advanced ovarian carcinoma. *Br J Obstet Gynaecol* **97**: 342–351

Meerpohl HG, Kuhnle J, Sauerbrei W, Achterrach W and Pfeiderer A (1990) Cyclophosphamide/cisplatin (CTX/PT) vs CTX/carboplatin (CarboPT) in advanced ovarian carcinoma: a randomised multicentre study. *J Cancer Res Clin Oncol* **16**: 1120

Omura G, Blessing JA, Ehrlich CE, Miller A, Yordan E, Creasman WT, et al (1986) A randomised trial of cyclophosphamide and doxorubicin with or without cisplatin in advanced ovarian carcinoma. A Gynecologic Oncology Group Study. *Cancer* **57**: 1725–1730

Ovarian Cancer Meta-analysis Project (1991) CP versus CAP chemotherapy of ovarian carcinoma: a meta-analysis. *J Clin Oncol* **9**: 1669–1679

Rankin EM, Mill L, Kaye SB, Atkinson R, Cassidy L, Cordiner J, et al (1992) A randomised study comparing standard dose carboplatin with chlorambucil and carboplatin in advanced ovarian cancer. *Br J Cancer* **65**: 275–281

Skarlos DV, Aravantinos G, Kosmidis P, Pavlidis N, Gennatas K, Beer M, et al (1996) Carbo alone compared with its combination with epirubicin and cyclophosphamide in untreated advanced epithelial ovarian cancer: a Hellenic Cooperative Oncology Study Group. *Eur J Cancer* **32**: 421–428

Sturgeon JFG, Fine S, Gospodarowicz MK, Dembo AJ, Bean HA, Bush RS et al (1982) A randomised trial of melphalan alone vs combination chemotherapy in advanced ovarian cancer. *Proc Am Soc Clin Oncol* **1**: (abstract 108)

Swenerton K, Jeffrey J, Stuart G, Roy M, Krepart G, Carmichael J, et al (1992) Cisplatin–cyclophosphamide versus carboplatin–cyclophosphamide in advanced ovarian cancer: a randomised phase III study of the National Cancer Institute of Canada Clinical Trials Group. *J Clin Oncol* **10**: 718–726

Taylor AE, Wiltshaw E, Gore M, Fryatt I and Fisher C (1994) Long-term follow up of the first randomised study of cisplatin versus carboplatin for advanced epithelial ovarian cancer. *J Clin Oncol* **12**: 2066–2070

Ten Bokkel Huinik WW, van der Burg ME, van Oosterom AT, Neijt JP, George M, Gustalla JP, et al (1988) Carboplatin in combination chemotherapy for ovarian cancer. *Cancer Treat Rev* **15**:(suppl.) 9–15

Tomirotti M, Perrone S, Gie P, Canaletti R, Carpi A, Biasoli R, et al (1988) Cisplatin (P) versus cyclophosphamide, adriamycin and cisplatin (CAP) for stage III–IV epithelial ovarian carcinoma: a prospective randomised trial. *Tumori* **74**: 573–577

Torri V on behalf of the International Ovarian Neoplasm Studies (1996) Randomised study of cyclophosphamide, doxorubicin and cisplatin (CAP) vs single-agent carboplatin in ovarian cancer patients requiring chemotherapy: interim results of ICON2. *Proc Am Soc Clin Oncol* **15**: 280 (abstract 752)

Trope C, Anderson H, Björkholm E, Frankendal B, Himmelman A, Högberg T, et al (1998) Doxorubicin–melphalan with and without cisplatin in advanced ovarian cancer. Ten-year survival results from a prospective randomised study by the Swedish Cooperative Ovarian Cancer Study Group (in press)

Wadler S, Yeap B, Vogl S and Carbone P (1996) Randomised trial of initial therapy with melphalan versus cisplatin-based combination chemotherapy in patients with advanced ovarian cancer: initial and long-term results: Eastern Cooperative Oncology Study Group E2878. *Cancer* **77**: 733–742

Wilbur DW, Rentschler RE, Wagner RJ, Keeney ED, King A and Hilliard DA (1987) Randomised trial of the addition of cisplatin (DDP) and/or BCG to cyclophosphamide (CTX) chemotherapy for ovarian carcinoma. *J Surg Oncol* **34**: 165–169

Williams CJ, Mead GM, Macbeth FR, Thompson J, Whitehouse JMA, MacDonald H, et al (1985) Cisplatin combination chemotherapy versus chlorambucil in advanced ovarian carcinoma: mature results of a randomised trial. *J Clin Oncol* **3**: 1455–1452

Wiltshaw E, Evans B, Rustin G, Gilbey E, Baker J and Barker G (1986) A prospective randomised trial comparing high-dose cisplatin with low-dose cisplatin and chlorambucil in advanced ovarian carcinoma. *J Clin Oncol* **4**: 722–729

Yusuf S, Peto R, Lewis J, Collins R and Sleight T (1985) Beta blockade during and after myocardial infarction: an overview of randomised clinical trials. *Prog Cardiov Dis* **27**: 335–371

### Unpublished

Crowther D (1996) A randomised trial of chemotherapy in advanced residual (stage lib–IV) ovarian cancer. Manchester Ovarian Cancer Clinical Study Group protocol. (Unpublished)

© Cancer Research Campaign 1998

Turbow MM (1980) Chemotherapy of advanced ovarian cancer: Adriamycin–cyclophosphamide versus platinum–Adriamycin–cyclophosphamide. Northern California Oncology Group protocol 5091. (Unpublished)

Gilby E, Pollard W, Bamford D, Barley V, Jelen I, Hale BT, et al. Ovarian Cancer Trial, 1986. Southwest Oncology Study. (Unpublished)

Medical Research Council Gynaecological Cancer Working Party. Inadequacy of trials of chemotherapy in advanced ovarian carcinoma: a randomised trial of three regimens. (Unpublished)

© Cancer Research Campaign 1998